# A Benzimidazole-Based Fluorescent Probe for the Selective Recognition of Cobalt (II) Ions

**DOI:** 10.3390/molecules30153309

**Published:** 2025-08-07

**Authors:** Jing Zhu, Hua-Fen Wang, Jia-Xiang Zhang, Man Wang, Yu-Wei Zhuang, Zhi-Guang Suo, Ye-Wu He, Yan-Chang Zhang, Min Wei, Hai-Yan Zhang

**Affiliations:** 1 Henan Key Laboratory of Cereal and Oil Food Safety Inspection and Control, College of Food Science and Technology, Henan University of Technology, Zhengzhou 450001, Chinawei_min80@163.com (M.W.); 2 High & New Technology Research Center of Henan Academy of Sciences, No. 56 Hongzhuan Road, Zhengzhou 450002, Chinahaiyanhai6828_cn@sina.com (H.-Y.Z.); 3 The Material Research Institute of Henan Academy of Sciences, Zhengzhou 450046, China

**Keywords:** fluorescent probe, cobalt ion recognition, benzimidazole-based

## Abstract

Cobalt, a rare element in the Earth’s crust, is widely used in industries due to its hardness and antioxidant properties. It also plays a vital role in physiological functions, being a key component of vitamin B_12_. However, excessive cobalt intake can cause health issues. Detecting cobalt ions, especially Co^2+^, in food is crucial due to potential contamination from various sources. Fluorescent probes offer high sensitivity, selectivity, a rapid response, and ease of use, making them ideal for the accurate and efficient recognition of Co^2+^ in complex samples. In this context, a highly selective fluorescent probe, 2,2′-((3-(1H-benzo[d]imidazol-2-yl)-1,2-phenylene) bis(oxy)) bis(N-(quinolin-8-yl) acetamide) (**DQBM-B**), was synthesized using chloroacetyl chloride, 8-aminoquinoline, 2,3-dihydroxybenzaldehyde, and benzidine as raw materials for the recognition of Co^2+^. Probe **DQBM-B** can exhibit fluorescence alone in DMF. However, as the concentration of Co^2+^ increased, Photoinduced Electron Transfer (PET) occurred, which quenched the original fluorescence of the probe. Probe **DQBM-B** shows better selectivity for Co^2+^ than other ions with high sensitivity (detection limit: 3.56 μmol L^−1^), and the reaction reaches equilibrium within 30 min.

## 1. Introduction

Cobalt is a rare element found in some parts of the Earth’s crust. Because of its hardness and antioxidants, it is used in different products and processes, for example, magnet and stainless steel alloys, batty manufacturing, pigments, automotive industries, metal finishing, mining, electric cable manufacturing, coloring and catalysts. Therefore, this element is an important metal in industries [1]. Cobalt is usually found in bivalent (Co^2+^, Co (II)) and trivalent (Co^3+^, Co (III)) states, while Co^2+^ and Co^3+^ are essential trace elements for plants and animals [2]. Cobalt also plays a vital role in numerous physiological functions [3]. For example, Co^2+^ is indispensable for humans as it constitutes an integral part of vitamin B_12_ [4,5]. Cobalt deficiency can lead to megaloblastic anemia and impinge on the development of the immune system [2,6]. However, excessive cobalt intake can affect the nervous, endocrine, and respiratory systems, leading to diseases such as thyroiditis and skin allergies [7,8]. Exposure to cobalt primarily stems from mining, incineration, cosmetics, diet, and medical treatments, among which diet is considered to be one of the most important sources of cobalt exposure [9,10,11,12]. Cobalt contamination in food may come from food processing, packaging or contaminated drinking water [13,14]. Given the widespread use of cobalt in various industrial and biological contexts, the accurate recognition of cobalt ions, especially Co^2+^, is crucial for both environmental and health safety.

The most commonly employed traditional detection methods for Co^2+^ include atomic absorption spectroscopy (AAS), energy-dispersive X-ray spectroscopy (EDS), and inductively coupled plasma mass spectrometry (ICP-MS) [15]. Although the above detection methods offer high sensitivity and accuracy in heavy metal detection, they demand the involvement of professional technical personnel and sample pretreatment. Presently, researchers are actively engaged in creating diverse chemosensors for the detection of Co^2+^, including electrochemical sensors, fluorescent sensors, and colorimetric sensors. Among these methods for identifying Co^2+^, colorimetry and fluorometry are considered the best options due to their user-friendliness, sensitivity, accuracy, linearity and robustness [15,16].

High selectivity is a key factor for the accurate recognition of target ions in complex matrices using fluorescent probes [17]. Given the striking similarities in ionic radii and charge between Co^2+^ (74.5 pm) and Cu^2+^ (73.0 pm), these metal ions engage in intense competition when binding to probe ligands, particularly those with nitrogen or oxygen donors. This competition often presents notable challenges for the selective recognition of Co^2+^ in the presence of Cu^2+^. Currently, many fluorescent probes for Co^2+^ detection may be affected by interference from Cu^2+^ and other substances, which can impact the accuracy of Co^2+^ detection to some extent. For instance, in the study by Çimen et al., the benzimidazole–benzothiadiazole derivative BI-T exhibited a significant fluorescence intensity decrease towards both Co^2+^ and Cu^2+^ in benzonitrile [18]; Baruah et al. reported that their (R)-(−)-4-phenyl-2-oxazolidone-based fluorescent probe showed an obvious fluorescence intensity decline in response to various divalent cations, including Co^2+^ and Cu^2+^ [19]; and in the study by Guo et al., the polythiophene derivative PTMA displayed significant fluorescence intensity reduction upon interacting with Co^2+^ and Cu^2+^ in MeCN/Tris-HCl solution [20]. These findings collectively indicate that Cu^2+^, along with other ions, can introduce interference in the detection of Co^2+^, posing challenges for specific sensing.

The **DQBM-B** probe in this work demonstrates superior selectivity, particularly showing minimal interference from common ions like Cu^2+^, which ensures a more accurate and reliable recognition of Co^2+^. Given the extremely low concentration of Co^2+^ in food samples and the complexity of the detection environment [21,22], it is crucial to develop highly selective probes to enhance the accuracy and sensitivity of detection [23]. At present, there are relatively few highly selective fluorescent probes available for Co^2+^ detection in food samples. Quinoline–benzimidazole derivatives are known for their ability to coordinate with metal ions [24,25], which can significantly improve detection selectivity [26]. The development of such highly selective probes is essential to meet the demand for the rapid, sensitive, and accurate detection of Co^2+^ in complex matrices, thereby ensuring the better monitoring and control of Co^2+^ levels in various applications.

**Scheme 1:**
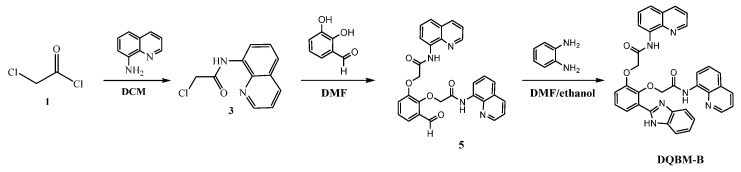
Synthetic scheme for **DQBM-B**.

In this work, we designed and synthesized a novel benzimidazole-based turn-off fluorescence probe **DQBM-B** for the recognition of Co^2+^ with high selectivity (Scheme 1). Probe **DQBM-B** exhibits fluorescence in a DMF solution, but as the Co^2+^ concentration increases, its fluorescence is quenched due the PET mechanism. The probe demonstrates high selectivity and anti-interference capabilities, with a detection limit of 3.56 μmol L^−1^, and the reaction reaches equilibrium within 30 min.

## 2. Results and Discussion

### 2.1. Stokes Shift of ***DQBM-B***

**Figure 1:**
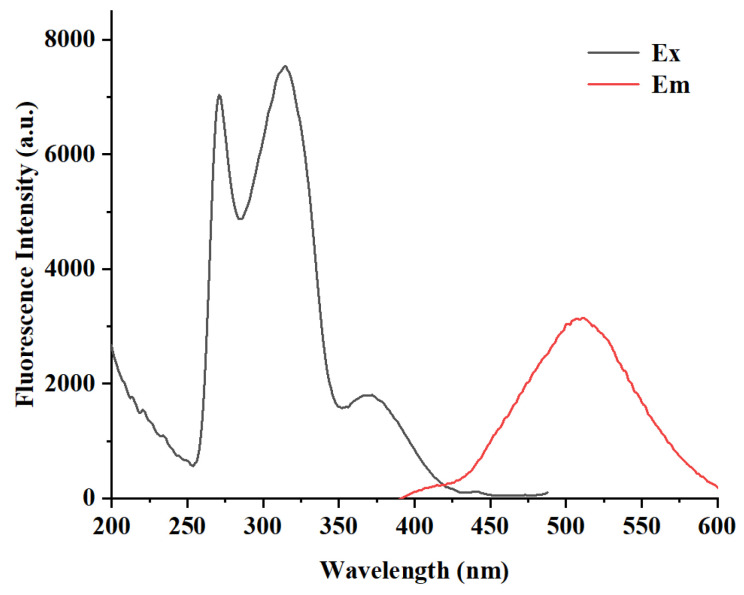
The excitation spectrum and the emission spectrum of **DQBM-B** in DMF (0.7 μmol L^−1^).

As depicted in Figure 1, probe **DQBM-B** demonstrates a remarkable Stokes shift of 137 nm. The excitation peak corresponding to the transition from S_0_ to S_1_ is located at 371 nm and fluorescence emission at 508 nm. This is significantly larger than the typical Stokes shift observed in many other probes [17]. The large Stokes shift effectively minimizes spectral overlap between the excitation and emission bands, thereby reducing background interference and self-quenching effects. This feature enables **DQBM-B** to maintain high recognition accuracy even in complex matrices, such as biological fluids or industrial wastewater. A Stokes shift of 137 nm enhances the signal-to-noise ratio, allowing for a more precise quantification of Co^2+^ in real-world samples.

### 2.2. Effect of Water Content on ***DQBM-B***

The water content represents a pivotal factor that exerts a significant impact on the fluorescent spectrum of the probe. Initially, the influence of water content in DMF on the fluorescence of **DQBM-B** was investigated. During the experiment, the concentration of **DQBM-B** was set at 0.7 μmol L^−1^.

**Figure 2:**
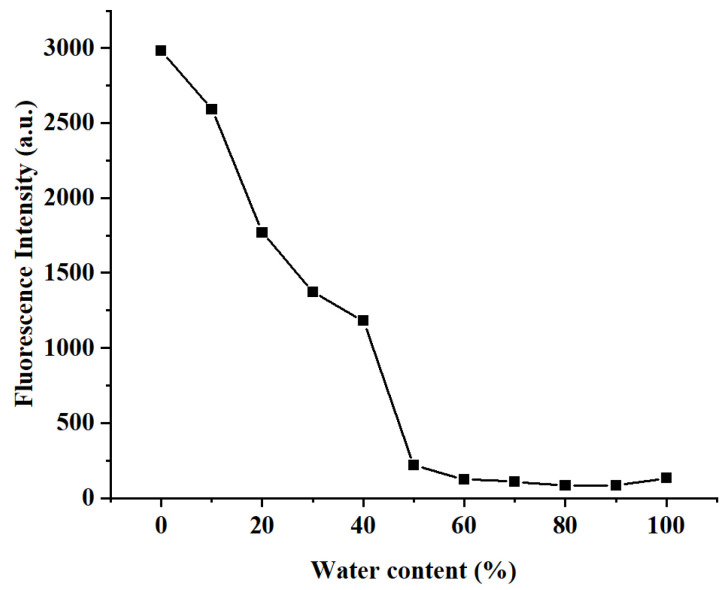
Effect of water content in DMF (0–100%) on fluorescence intensity of **DQBM-B** (0.7 μmol L^−1^). (λex = 308 nm, λem = 508 nm).

The result is shown in Figure 2. It can be seen that with the increase in water content in DMF, the fluorescence intensity of **DQBM-B** decreased rapidly. When the water content was more than 50% (*v*/*v*), it caused the complete fluorescence quenching of **DQBM-B**. This may be attributed to the Aggregation-Caused Quenching (ACQ) effect [27,28].

The results demonstrate that the water content of the solvent significantly affects the fluorescence properties of **DQBM-B**. Therefore, in order to reduce the influence of water content on fluorescence intensity, the water content of the solvent was uniformly set at 5% in subsequent experiments.

### 2.3. Stability and Ambient Light Effect on Fluorescence Emission of ***DQBM-B***

**Figure 3:**
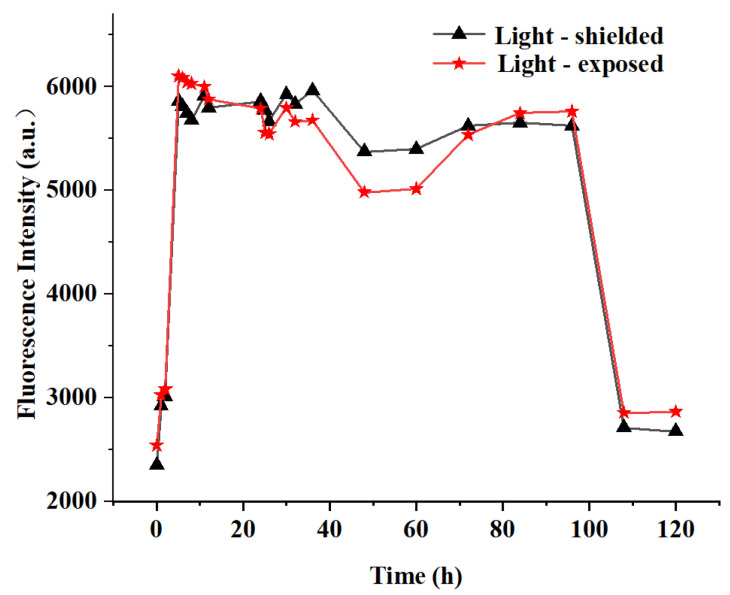
The change in fluorescence intensity of **DQBM-B** (0.7 μmol L^−1^) with time in DMF in the presence of natural light and in the absence of natural light. (λex = 308 nm, λem = 508 nm).

The stability and the influence of ambient light on the fluorescence emission of **DQBM-B** were investigated. As shown in Figure 3, the fluorescence intensity change in **DQBM-B** (0.7 μmol L^−1^) was monitored over time under different conditions.

Initially, the stability of **DQBM-B** was examined. As shown in Figure 3, the intensity remained stable within the first four hours, with minimal variation observed. However, an obvious fluorescent signal enhancement was observed from the fifth hour, and this phenomenon might be caused by the solvent effect [29]. Over the subsequent 96 h period, the fluorescence intensity remained virtually constant, demonstrating excellent long-term stability. At 108 h, a rapid decline in fluorescence intensity occurred, which might be induced by ACQ. This suggests that after prolonged exposure, the probe may undergo aggregation, leading to the observed quenching.

Subsequently, the effect of ambient light on the fluorescence of **DQBM-B** was examined to determine the optimal sensing conditions. Figure 3 shows that there was no significant difference in the trend of fluorescence intensity changes under both ambient light and dark conditions, indicating that **DQBM-B** exhibits good stability under natural light. Thus, the presence of ambient light does not significantly affect its fluorescence performance. This finding is crucial for practical applications, as it ensures that the probe can be used in environments with varying light conditions without compromising its performance.

In summary, **DQBM-B** shows good stability over a long-term period with a slight fluorescence intensity change in the first 4 h and a stable fluorescence intensity for up to 96 h. Additionally, it maintains its fluorescence stability under natural light and dark conditions, making it suitable for sensing applications in various environments.

### 2.4. Fluorescence Emission Changes with Different ***DQBM-B*** Concentrations

**Figure 4:**
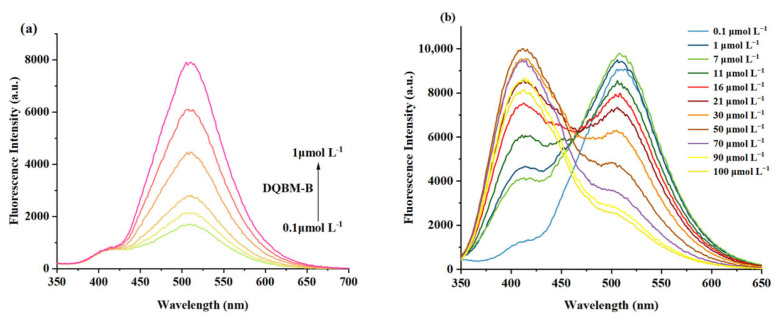
(**a**) The fluorescence spectra of **DQBM-B** at different concentrations (0.1–1 μmol L^−1^) in DMF (λex = 308 nm) (**b**) The fluorescence spectra of **DQBM-B** at different concentrations (0.1–100 μmol L^−1^) in DMF (λex = 308 nm).

With an increase in **DQBM-B** concentration from 0.1 μmol L^−1^ to 1 μmol L^−1^, the fluorescence spectrum of the probe exhibited only a single peak at 508 nm, and the fluorescence intensity increased progressively (Figure 4a). Interestingly, as can be seen in Figure 4b, when the probe concentration increased to 1 μmol L^−1^, **DQBM-B** exhibited two emission peaks at about 410 nm and 508 nm. With the continued increase in **DQBM-B** concentration, the emission peak (at 508 nm) declined gradually, while the other emission peak (at 410 nm) accordingly enhanced. This phenomenon suggests that the fluorescence behavior of **DQBM-B** is highly dependent on its concentration in solution. At lower concentrations, the probe’s molecules are likely to exist predominantly in a monomeric form, which accounts for the single emission peak at 508 nm. As the concentration increases, the likelihood of molecular interactions, such as aggregation or dimerization, becomes more significant. This is evidenced by the emergence of the second emission peak at 410 nm, which can be attributed to the formation of dimers of **DQBM-B**. The gradual increase in the intensity of the 410 nm peak with a further concentration increase indicates that the dimeric form predominates over the monomeric form in highly concentrated probe solutions. The bimodal fluorescence spectra of probe molecules at medium concentration may be due to the coexistence of dimers and monomers [30]. When the concentration continued to increase, the number of dimers increased while that of monomers decreased, resulting in the disappearance of the peak at 508 nm.

### 2.5. Fluorescence Emission Changes in ***DQBM-B*** Towards Co^2+^

**Figure 5:**
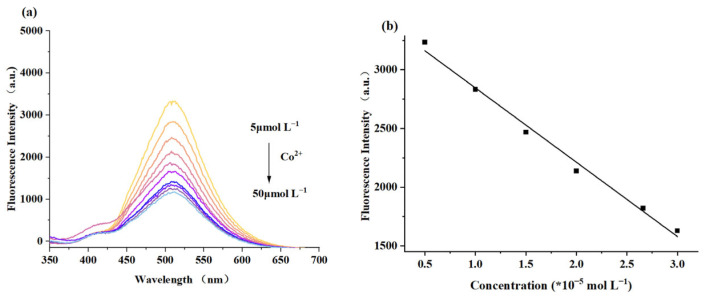
(**a**) The fluorescence spectra of **DQBM-B** (0.7 μmol L^−1^) with different concentrations of Co^2+^ in DMF (with 5% water content *v*/*v*) (λex = 308 nm). (**b**) The variation in the fluorescence intensity (508 nm) corresponds to the concentration of Co^2+^ in DMF (with 5% water content *v*/*v*) (the solid black line is the linear fitting line) (λex = 308 nm, λem = 508 nm).

The detecting feasibility of this probe was investigated by fluorescence titration experiments. Obviously, the fluorescence intensity of **DQBM-B** exhibited a gradual decrease upon the addition of Co^2+^ (Figure 5a). When Co^2+^ concentration was increased from 0.5 to 5.0 μmol L^−1^, the fluorescent intensity of **DQBM-B** (0.7 μmol L^−1^) gradually decreased from 3234.24 to 1159.65. This significant change in fluorescence intensity demonstrates the high sensitivity of **DQBM-B** towards Co^2+^ ions, indicating its potential as an effective sensor for recognizing Co^2+^ in solution.

A linear relationship was also found between the fluorescence intensity at 508 nm and the concentration of Co^2+^ over a range of 5–30 μmol L^−1^ (R = 0.988, Figure 5b). Based on the fluorescence titration measurement, the limit of detection (LOD) of **DQBM-B** for Co^2+^ was calculated to be 3.56 μmol L^−1^ according to the equation [31].

(1) $$LOD=\frac{3\sigma }{S},$$

(*σ*: the deviation of the response value and *S*: the slope of the standard curve).

This indicates that sensor **DQBM-B** can be developed as a highly selective and sensitive fluorescence chemosensor for recognizing Co^2+^ with a low detection limit.

### 2.6. Response Time

**Figure 6:**
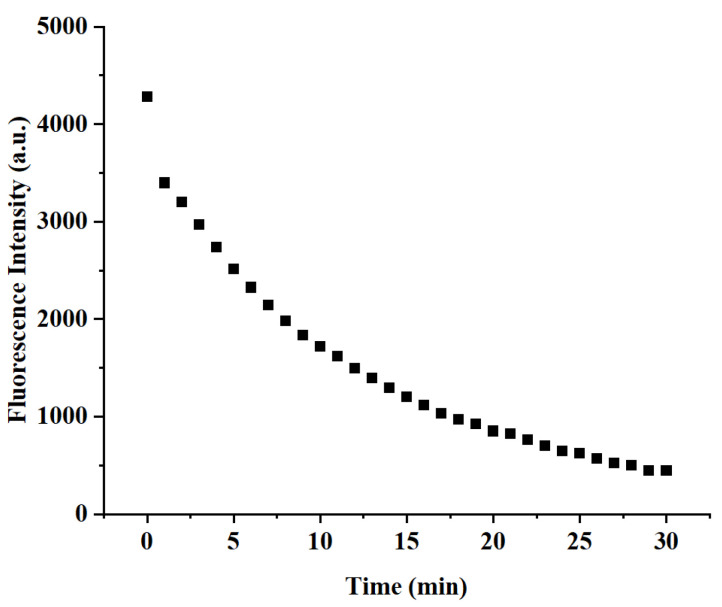
Time-dependent changes in the fluorescence intensity of **DQBM-B** (0.7 μmol L^−1^) in the presence of 2 equiv. Co^2+^ in DMF (with 5% water content *v*/*v*). (λex = 308 nm, λem = 508 nm).

In addition to detection limit, response time is another very important factor for probes. Therefore, the change in fluorescence intensity of **DQBM-B** over time after adding two equivalents of Co^2+^ was investigated, and the result is shown in Figure 6. It can be seen that the fluorescence intensity at 508 nm continued to decrease before the 29th minute and then remained almost constant. This indicates that the interaction between **DQBM-B** and Co^2+^ reaches equilibrium within approximately 30 min. To ensure enough time for the chelation of **DQBM-B** and Co^2+^, all measurements were carried out after maintaining the mixture solution of **DQBM-B** and metal ions for 30 min.

### 2.7. Selectivity and Anti-Interference Capacity of ***DQBM-B***

**Figure 7:**
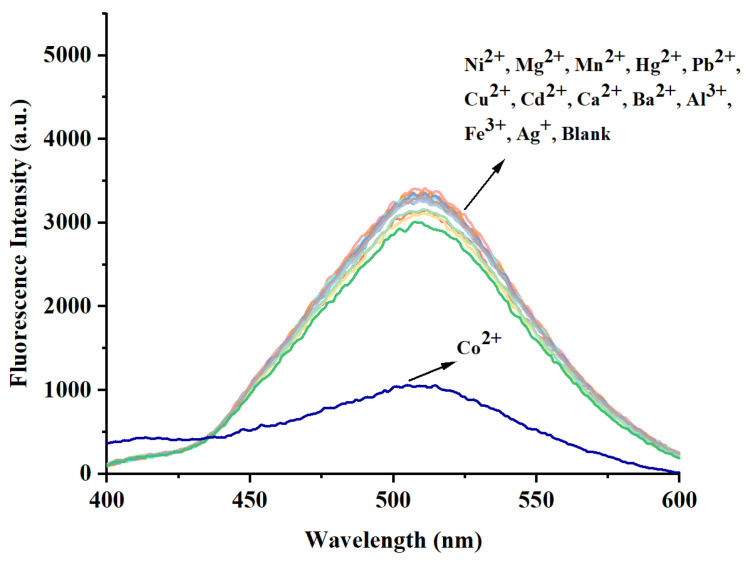
Fluorescence spectra of **DQBM-B** (0.7 μmol L^−1^) in the presence of Co^2+^ and other metal ions in DMF (with 5% water content *v*/*v*) (λex = 308 nm).

To evaluate the sensing properties of **DQBM-B** toward different metal ions, a selectivity experiment was conducted (Figure 7). Solutions of various metal ions, including Ba^2+^, Ca^2+^, Al^3+^, Co^2+^, Fe^3+^, Hg^2+^, Cd^2+^, Mn^2+^, Mg^2+^, Ni^2+^, Cu^2+^, Pb^2+^ and Ag^+^, were used to investigate the ion selectivity of probe **DQBM-B**. Two equivalents of each metal ion were added to the probe solution, and the fluorescence spectra of the resultant mixtures were recorded 30 min after the addition.

As shown in Figure 7, upon the addition of two equivalents of different metal ions, only Co^2+^ induced a distinct spectral change, while other metal ions showed almost no changes in the fluorescence spectra relative to **DQBM-B**. The fluorescence intensity of the probe decreased significantly after adding Co^2+^ (Figure 7).

In summary, the addition of Co^2+^ induces a significant change in fluorescence intensity, possibly attributed to the PET effect. This result implies that fluorescence probe **DQBM-B** has high selectivity for Co^2+^ over common metal ions.

**Figure 8:**
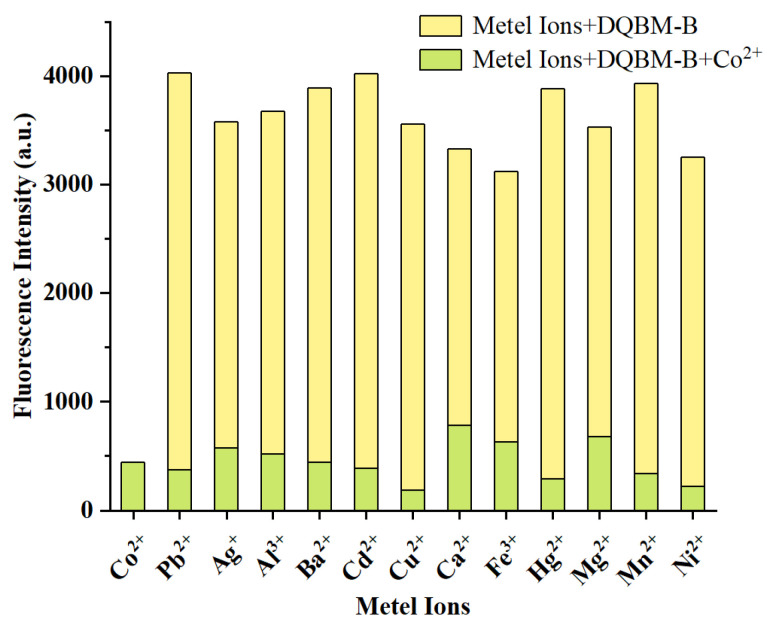
Fluorescence intensity of probe **DQBM-B** with Co^2+^ in the absence and presence of 2 equiv. of other metals ions in DMF (with 5% water content *v*/*v*) (λex = 308 nm, λem = 508 nm).

To further evaluate the performance of **DQBM-B** as a Co^2+^ probe in the presence of other metal ions, the anti-interference capability of the **DQBM-B**-Co^2+^ complex was assessed. As shown in Figure 8, in the presence of other metal ions, fluorescent quenching induced by Co^2+^ showed negligible changes compared with the blank sample.

This experiment was designed to simulate real-world conditions where the probe might encounter a mixture of metal ions. The results indicate that the presence of other metal ions does not interfere with the specific interaction between **DQBM-B** and Co^2+^, further confirming its robustness and reliability as a selective sensor. Overall, these findings highlight the potential of **DQBM-B** as a highly selective and sensitive fluorescence chemosensor for Co^2+^, suitable for applications in complex environments.

### 2.8. Job’s Plot

**Figure 9:**
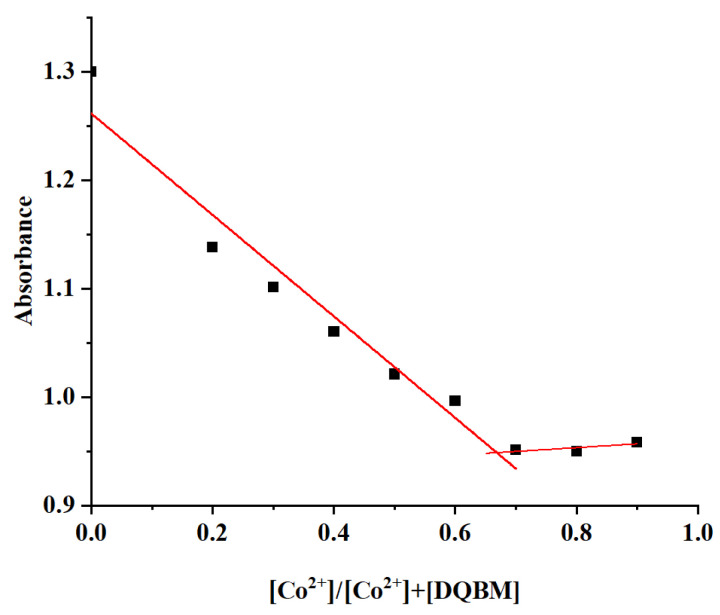
Job’s plot for **DQBM-B** + Co^2+^ complex in DMF (with 5% water content *v*/*v*).

The coordination stoichiometry of the complex formed between **DQBM-B** and Co^2+^ was investigated by Job’s plot measurements [32]. As shown in Figure 9, there is a continuous decline in absorbance when the molar ratio of Co^2+^ to **DQBM-B** falls below 2. However, once the molar ratio of Co^2+^ to **DQBM-B** reaches 2, the absorbance stabilizes and shows negligible change. This revealed that the stoichiometric ratio was 2:1 in the Co^2+^ complex. This 2:1 ratio suggests that each **DQBM-B** molecule coordinates with two Co^2+^ ions, forming a stable complex. This coordination behavior is crucial for understanding the interaction mechanism between **DQBM-B** and Co^2+^, and it provides important insights into the design of **DQBM-B** as a selective sensor for Co^2+^.

### 2.9. Recognition Mechanism

**Figure 10:**
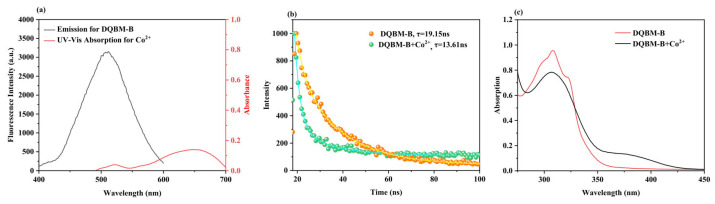
(**a**) The fluorescence emission spectrum of **DQBM-B** in DMF (with 5% water content *v*/*v*) (λex = 308 nm) and the absorption spectrum of Co^2+^ in DMF (with 5% water content *v*/*v*). (**b**) Fluorescence lifetimes of **DQBM-B** and **DQBM-B** with added Co^2+^ in DMF (with 5% water content *v*/*v*). (**c**) UV absorption spectra of **DQBM-B** and **DQBM-B** with added Co^2+^ in DMF (with 5% water content *v*/*v*).

In Figure 10, the quenching mechanism of probe **DQBM-B** for Co^2+^ is explored. As shown in Figure 10a, the emission spectrum of **DQBM-B** in DMF (with 5% water content *v*/*v*) (λex = 308 nm) has almost no significant overlap with the absorption spectrum of Co^2+^ in DMF (with 5% water content *v*/*v*). Moreover, the absorption intensity of cobalt chloride in DMF (with 5% water content *v*/*v*) is very weak. Therefore, Förster resonance energy transfer (FRET) and the inner filter effect (IFE) can be ruled out. Next, we tested and calculated the average fluorescence lifetimes of **DQBM-B** and **DQBM-B** with added Co^2+^ (Figure 10b). The fluorescence lifetime data were fitted using an exponential model (the residual value x^2^ ≤ 1.2).

(2) $$R\left(t\right)=\sum_{i}B_{i}e^{-\frac{t}{\tau_{i}}}$$

*R*(*t*): the fluorescence intensity at time *t*; *B_i_*: the pre-exponential factor of the *i*-th term at time *t*; and *τ_i_*: the fluorescence lifetime of the *i*-th term.

The average fluorescence lifetime is calculated using the following formula:

(3) $$\tau_{av}=\frac{\sum \alpha_{i}\tau_{i}^{2}}{\sum \alpha_{i}\tau_{i}}$$

*α_i_*: the proportion of lifetime *τ_i_*.

The results show that after the addition of Co^2+^, the average fluorescence lifetime of the probe decreased from 19.15 ns to 13.61 ns, which is consistent with the characteristics of dynamic quenching. As shown in Figure 10c, after the addition of Co^2+^ to **DQBM-B**, the UV absorption peak intensity at 308 nm decreased, and a new absorption peak appeared in the range of 350–400 nm. Therefore, it can be concluded that after the addition of Co^2+^, the PET effect occurred, resulting in fluorescence quenching.

**Figure 11:**
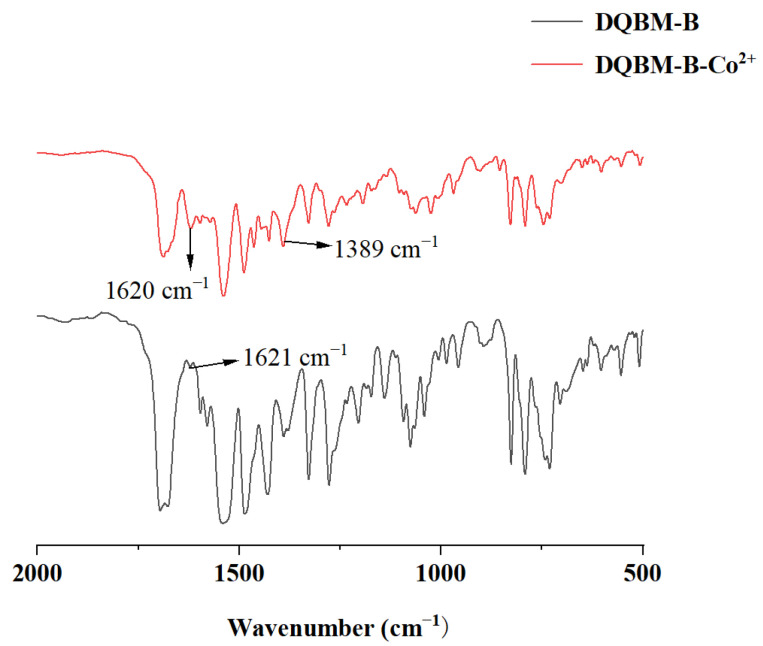
FT-IR spectra of **DQBM-B** before (black) and after (red) the addition of Co^2+^.

In order to delve deeper into the potential binding modes between **DQBM-B** and Co^2+^, FT-IR spectra were utilized to elucidate the coordination mechanism. FT-IR spectra of **DQBM-B** in the absence and presence of Co^2+^ are shown in Figure 11. Upon the addition of Co^2+^, the peak intensity at 1621 cm^−1^ increased, likely due to changes in the vibration mode of the C=N bond caused by the coordination interaction. Additionally, a new absorption peak at 1389 cm^−1^ emerged, likely originating from the altered electronic environment of the probe following the introduction of Co^2+^. This change may induce a shift in the C-N vibration frequency, thereby giving rise to the observed peak. These changes could demonstrate that a coordination bond has formed between Co^2+^ and probe **DQBM-B**.

**Figure 12:**
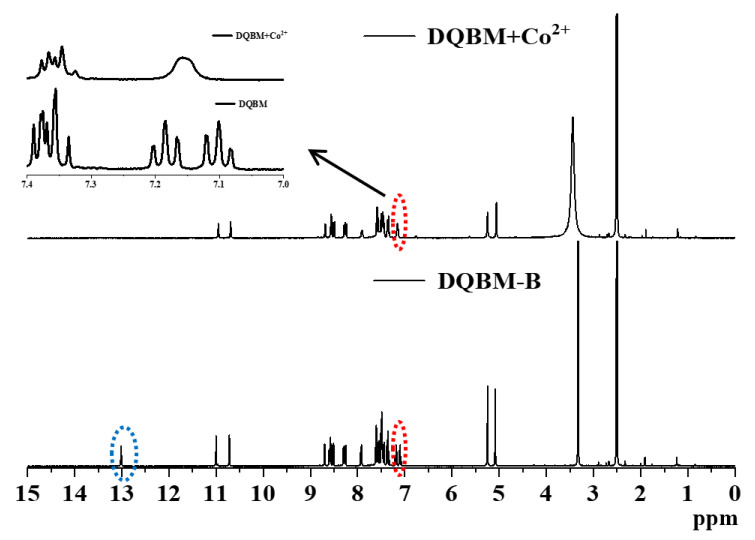
^1^H NMR of **DQBM-B** before and after addition of Co^2+^ in DMF (with 5% water content *v*/*v*).

^1^H NMR was also performed to demonstrate the interaction between the probe and the analyte (Figure 12). After the addition of Co^2+^, the proton peak on the imidazole ring disappears, and the two triple peaks (δ7.08, 7.16) on the benzene ring become single peaks, which could prove that a coordination bond between nitrogen and Co^2+^ on the imidazole ring is formed. Thus, there is an interaction between probe **DQBM-B** and Co^2+^. These ^1^H NMR observations provide additional evidence for the formation of coordination bonds between **DQBM-B** and Co^2+^.

The absence of spectral overlap between the emission spectrum of the probe and the absorption spectrum of Co^2+^ indicates that the quenching mechanism is not due to direct energy transfer. The decrease in fluorescence lifetime and the changes in the ultraviolet absorption peak collectively confirmed that the electron transfer process occurred upon the addition of Co^2+^, leading to fluorescence quenching. Additionally, the combined results from infrared spectroscopy and ^1^H NMR data demonstrated that Co^2+^ formed coordination bonds with probe **DQBM-B**. After the amine forms a coordination bond with Co^2+^, the electron cloud density decreases and the electron-withdrawing ability increases. This coordination interaction provided a structural basis for the PET process.

## 3. Materials and Methods

### 3.1. Reagents

8-aminoquinoline, chloroacetyl chloride, 2,3-Dihydroxybenzaldehyde, 1,2-diaminobenzene and all organic solvents were of analytical grade and purchased from Sinopharm Chemical Reagents Co. (Shanghai, China). The solutions of metal cations were prepared from their chlorides (Ba^2+^, Al^3+^, Cd^2+^, Ca^2+^, Zn^2+^, Co^2+^, Hg^2+^, and Fe^3+^), sulfates (Mn^2+^, Mg^2+^, Cu^2+^, and Ni^2+^) or nitrates (Pb^2+^ and Ag^+^).

### 3.2. Structural Characterizations

^1^H NMR and ^13^C NMR spectra are recorded by an NMR spectrometer (Agilent 400-MR, Agilent Technologies, Santa Clara, CA, USA). MS spectra are measured by a mass spectrometer (Agilent 6545 LC/Q-TOF, Agilent, Waltham, MA, USA). FT-IR spectra are recorded on an infrared spectrometer (Nicolet 6700, Thermo Fisher Scientific, Madison, WI, USA). UV-vis spectra are recorded on a UV-vis spectrophotometer (UV-1800, Mapada, Shanghai, China). The fluorescence intensities are monitored using a fluorescence spectrometer (FP-8550, Hitachi, Tokyo, Japan).

### 3.3. Synthesis of Compound **3**

8-Aminoquinoline (0.60 g, Compound **2**) and 30 mL of dichloromethane were added to a round-bottomed flask. The mixture was stirred at room temperature (25 °C) until it dissolved. Subsequently, 0.60 g of triethylamine and 0.70 g of chloroacetyl chloride (Compound **1**) were added sequentially. The reaction mixture was stirred at room temperature for 3 h. Then, a saturated potassium carbonate aqueous solution was added to the reaction mixture and stirred to remove excess chloroacetyl chloride. The organic phase was separated using a liquid separation funnel and then dried over anhydrous magnesium sulfate. The solvent was removed by rotary evaporation to obtain the crude product, which was further recrystallized from anhydrous ethanol to yield a white solid product with a yield of 89%.

^1^H NMR (Dimethylsulfoxide-D_6_): (δ; ppm) = 4.31 (s, 1H); 7.55 (s, 1H); 7.56 (s, 2H); 8.18 (d, 1H); 8.75 (q, 1H); 8.86 (q, 1H); 10.91 (s, 1H); ^13^C NMR (Dimethylsulfoxide-D_6_): (δ; ppm) = 43.28; 116.50; 121.69; 122.43; 127.04; 127.81; 136.16; 133.44; 138.59; 148.57; 164.26. MS: *m*/*z* calcd for C_11_H_9_C_l_N_2_O, 220; found 221 [M + H]^+^ (Figures S1–S3). The FT-IR spectrum of Compound **3** is shown in Figure S4, and the presence of the amide bond confirms the successful synthesis of Compound **3**.

### 3.4. Synthesis of Compound **5**

In total, 1.00 g of Compound **3**, 2.50 g of Compound **4**, 2.50 g of anhydrous potassium carbonate, and 40 mL of N, N-dimethylformamide (DMF) were added to a three-necked flask. The reaction mixture was heated to 85 °C for 2 h, while the progress of the reaction was monitored by TLC (the developing agent was a mixture of ethyl acetate and petroleum ether in a volume ratio of 1:4). Once the reaction was complete, the resulting mixture was cooled to room temperature and then poured into 80 mL of water in the three-necked flask. The mixture was allowed to crystallize at room temperature for 2.5 h and then filtered. The filter cake was washed with water and dried at 55 °C. The black solid crude product (containing Compound **5**) weighing 0.80 g was obtained and used for the next reaction.

^1^H NMR (Dimethylsulfoxide-D_6_): (δ; ppm) = 10.92 (s, 1H); 10.65 (s, 1H); 10.59 (s, 1H); 8.79 (q, 1H); 8.72 (q, 1H); 8.53 (t, 2H); 8.36 (m, 2H); 7.61 (m, 4H); 7.49 (m, 4H); 7.35 (t, 1H); 5.21 (s, 1H); 5.06 (s, 1H); ^13^C NMR (Dimethylsulfoxide-D_6_): (δ; ppm) = 190; 166.75; 166.75; 160.01; 150.51; 149.32; 149.05; 137.70; 136.50; 133.35; 129.76; 127.70; 126. 80; 125.30; 122.20; 120.50; 116.00; 73.06; 68.27. MS: *m*/*z* calcd for C_29_H_22_N_4_O_5_, 506.16; found 507.16 [M + H]^+^ (Figures S5–57). The infrared spectrum of Compound **5** is shown in Figure S8.

### 3.5. Synthesis of **DQBM-B**

In total, 0.80 g of the black solid crude product (containing Compound **5**), 0.67 g of sodium bisulfite, 25 mL of anhydrous ethanol, and 25 mL of N, N-dimethylformamide (DMF) were added to a three-necked flask. The mixture was stirred at room temperature for 12 h. Subsequently, 0.51 g of o-phenylenediamine (Compound **6**) was added. The reaction mixture was then heated to 85 °C for 3 h, while the progress of the reaction was monitored by TLC (the developing agent was a mixture of ethyl acetate and petroleum ether in a volume ratio of 1:1). Once the reaction was complete, the mixture was cooled to room temperature and the resulting mixture was slowly added to 50 mL of water in the three-necked flask. The mixture was allowed to crystallize at room temperature for 2.5 h and then filtered. The filter cake was washed with water and dried at 55 °C to yield 0.70 g of the black solid crude product. This crude product was purified by TLC (the developing agent was a mixture of methanol and dichloromethane in a volume ratio of 1:20) to obtain **DQBM-B** (2,2′-((3-(1H-benzo[d]imidazol-2-yl)-1,2-phenylene) bis(oxy)) bis(N-(quinolin-8-yl) acetamide)) with a yield of 68%.

^1^H NMR (Dimethylsulfoxide-D_6_): (δ; ppm) = 13.03 (s, 1H); 10.99 (s, 1H); 10.70 (s, 2H); 8.68 (d, 1H); 8.56–8.48 (q, 1H); 8.28 (d, 1H); 7.92 (s, 1H); 7.6–7.31 (s, 1H); 7.16 (s, 1H); 7.08 (s, 2H); 5.28 (d, 1H); 5.09 (q, 1H); ^13^C NMR (Dimethylsulfoxide-D_6_): (δ; ppm) = 167.9; 165.96; 152.7; 148.74; 142.99; 136.37; 134.62; 133.27; 127.60–121.73; 118.76–116.08; 111.62; 73.08; 68.50. MS: *m*/*z* calcd for C_35_H_26_N_6_O_4_, 594.20; found 595.20 [M + H]^+^ (Figures S9 and S10).

## 4. Conclusions

In conclusion, a new benzimidazole-based fluorescent probe (**DQBM-B**) for Co^2+^ recognition has been developed. The sensor is able to selectively recognize Co^2+^ by forming a 1:2 complex between **DQBM-B** and Co^2+^, which causes a fluorescence intensity decrease at 508 nm due to the PET effect upon Co^2+^ addition. The probe can also selectively recognize Co^2+^ in the presence of other interfering metal ions including but not limited to Fe^3+^, Ni^2+^, and Cu^2+^. A linear relationship was also found with the concentration of Co^2+^ of 5–30 μmol L^−1^ with a detection limit of 3.56 μmol L^−1^. This study provides new insights into the design and synthesis of Co^2+^ chemical sensors. This innovative probe not only addresses the limitations of existing detection methods but also offers a practical solution for the rapid and accurate recognition of Co^2+^ in complex samples, making it a valuable tool for environmental monitoring, food safety, and biomedical applications. For ACQ probes, it is expected that future efforts will focus on combining them with nanomaterials to improve the ACQ effect, thereby enhancing the practical application potential of the probes.

## Data Availability

The data can be obtained from the author.

## Supplementary Materials

The following supporting information can be downloaded at https://www.mdpi.com/article/10.3390/molecules30153309/s1, Figure S1: The ^1^H NMR spectrum of Compound **3**. Figure S2: The ^13^C NMR spectrum of Compound **3**. Figure S3: The mass spectrum of Compound **3**. Figure S4: The infrared spectrum of Compound **3**. Figure S5: The ^1^H NMR spectrum of Compound **5**. Figure S6: The ^13^C NMR spectrum of Compound **5**. Figure S7: The mass spectrum of Compound **5** (ESI^+^). Figure S8: The infrared spectrum of Compound **5**. Figure S9: The ^13^C NMR spectrum of **DQBM-B**. Figure S10: The mass spectrum of **DQBM-B**.

## Abbreviations

The following abbreviations are used in this manuscript:

**DQBM-B**	2,2′-((3-(1H-benzo[d]imidazol-2-yl)-1,2-phenylene)bis(oxy))bis(N-(quinolin-8-yl)acetamide)
PET	Photoinduced Electron Transfer
ACQ	Aggregation-Caused Quenching
