# Supplementary material for: A Benzimidazole-Based Fluorescent Probe for the Selective Recognition of Cobalt (II) Ions
Source: Molecules. 2025 Aug 7;30(15):3309. doi: 10.3390/molecules30153309 (PMC12348960; doi:10.3390/molecules30153309)
Supplement: Supplementary file 1 [file molecules-30-03309-s001.zip › molecules-3733712-supplementary.pdf]

Supporting Information

A Benzimidazole-Based Fluorescent Probe for the Selective Recognition of Cobalt (II) Ions

Jing Zhu [1,2,†], Hua-Fen Wang [1,2,*,†], Jia-Xiang Zhang [2,3], Man Wang [2,*], Yu-Wei Zhuang [2], Zhi-Guang Suo [1,*], Ye-Wu He [1,2], Yan-Chang Zhang [2], Min Wei [1] and Hai-Yan Zhang [2]

[1] Henan Key Laboratory of Cereal and Oil Food Safety Inspection and Control, College of Food Science and Technology, Henan University of Technology, Zhengzhou 450001, China; zjlsl7443@hnas.ac.cn (J.Z.); hyw213831@hnas.ac.cn (Y.-W.H.); wei_min80@163.com (M.W.)

[2] High & New Technology Research Center of Henan Academy of Sciences, No. 56 Hongzhuan Road, Zhengzhou 450002, China; zjx865185@hnas.ac.cn (J.-X.Z.); zyw0218@hnas.ac.cn (Y.-W.Z.); zyc@hnas.ac.cn (Y.-C.Z.); haiyanhai6828_cn@sina.com (H.-Y.Z.)

[3] The Material Research Institute of Henan Academy of Sciences, Zhengzhou 450046, China

* Correspondence: wanghuafen_2025@hnas.ac.cn (H.-F.W.); manmanwang202309@hnas.ac.cn (M.W.); zg_suo@163.com (Z.-G.S.)

† These authors contributed equally to this work.

Figure S1 is the ^{1}H NMR spectrum of compound **3**, Figure S2 is the ^{13}C NMR spectrum of compound **3**. Figure S3 is the mass spectrum of compound **3**, which shows a molecular weight of 220 for compound **3**, with a molecular ion peak at 221.Figure S4 is the infrared spectrum of compound **3**.

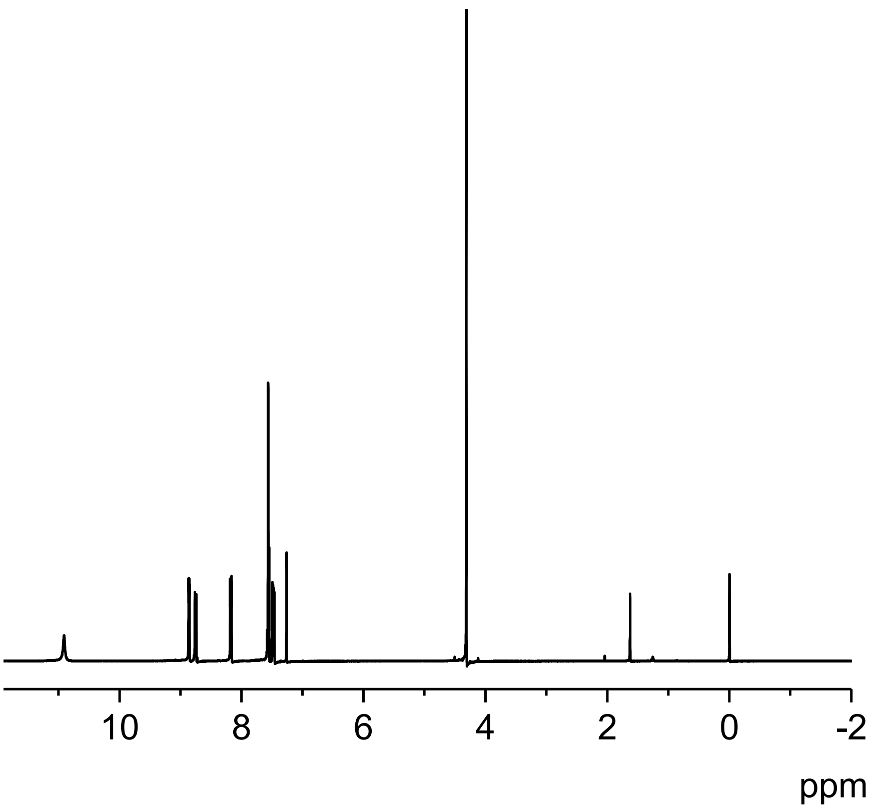

Figure S1 ^{1}H NMR spectrum of compound **3**.

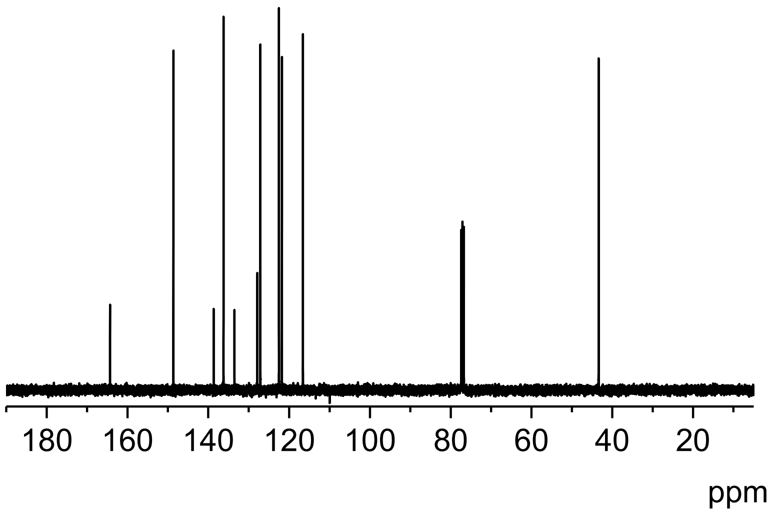

Figure S2 ^{13}C NMR spectrum of compound **3**.

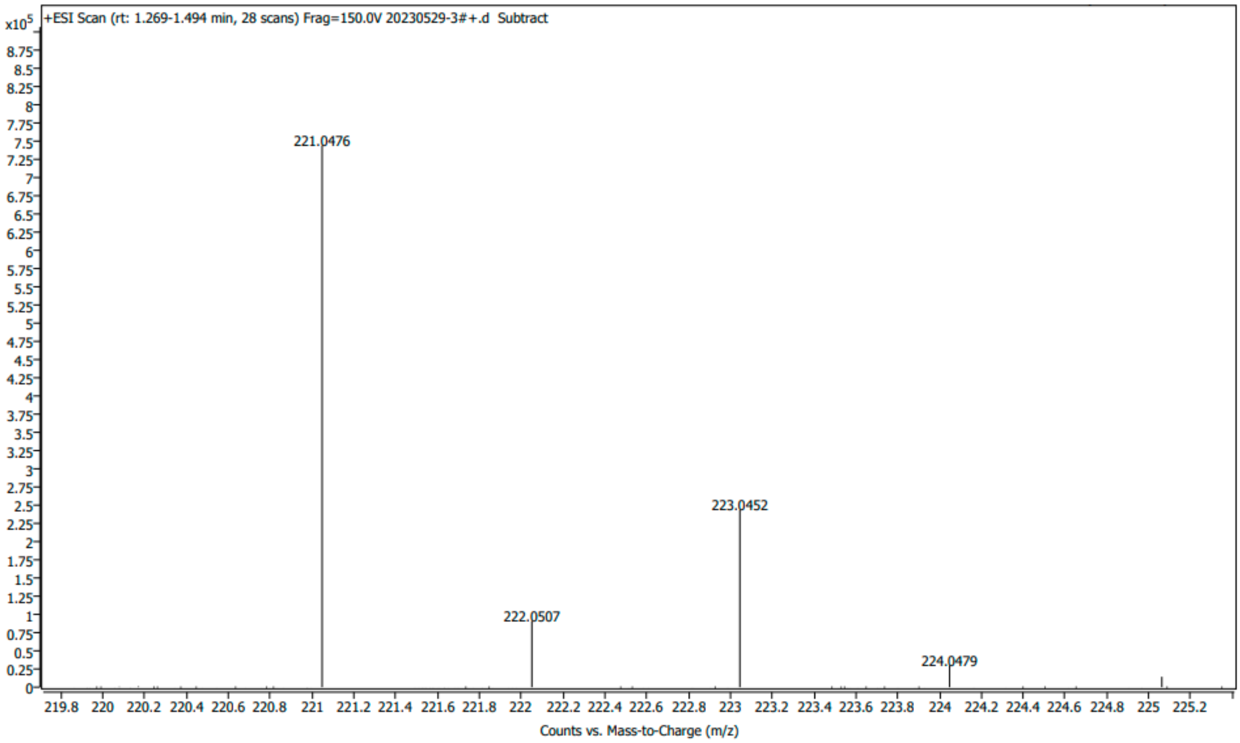

Figure S3 ESI mass spectrum of compound **3**.

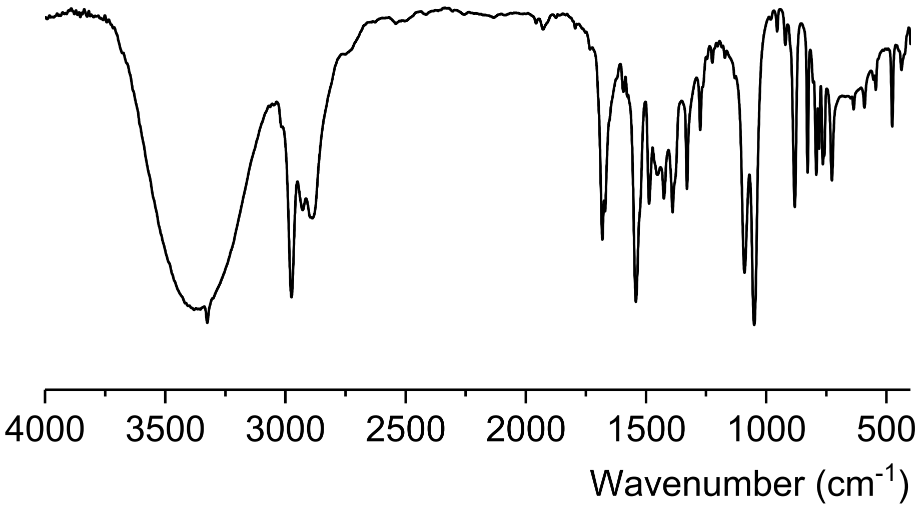

Figure S4 Infrared spectrum of compound **3**.

Figure S5 is the ^{1}H NMR spectrum of compound **5**. Figure S6 is the ^{13}C NMR spectrum of compound **5**. Figure S7 is the mass spectrum of compound **5** (ESI^{+}). The results show that the peak at 507.1666 corresponds to the $[M+H]^{+}$ ion of compound 5. Figure S8 is the infrared spectrum of compound **5**.

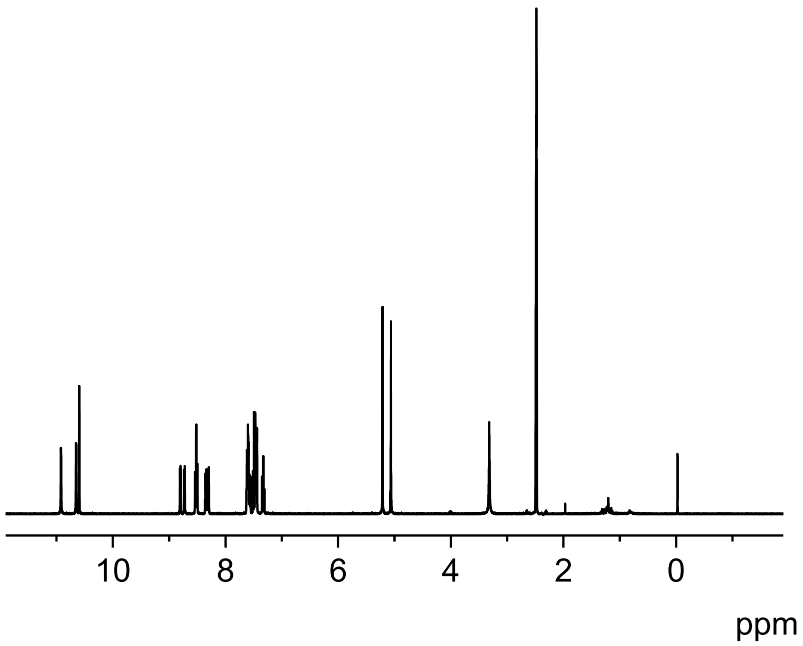

Figure S5 ^{1}H NMR spectrum of compound **5**.

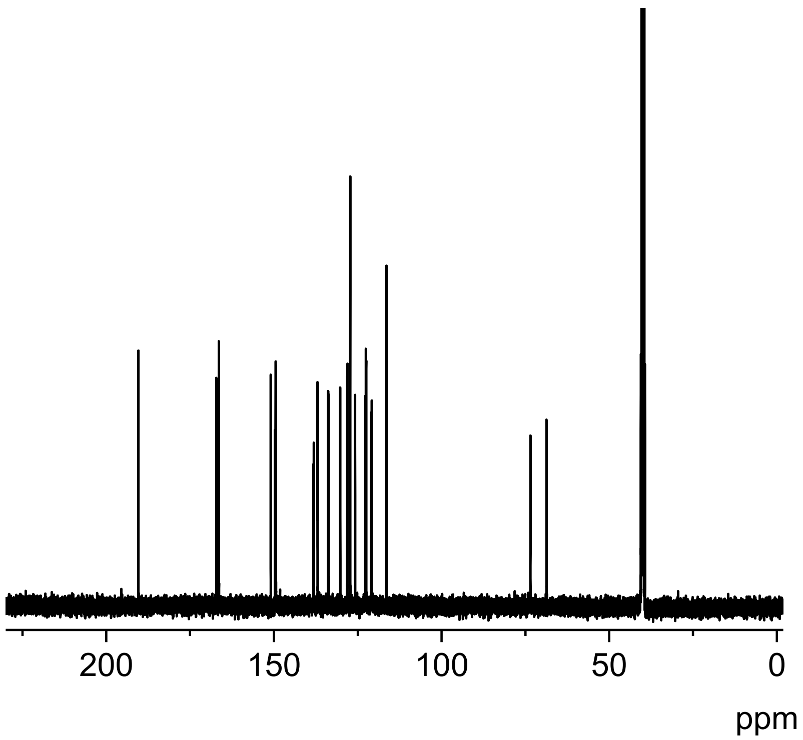

Figure S6 ^{13}C NMR spectrum of compound **5**.

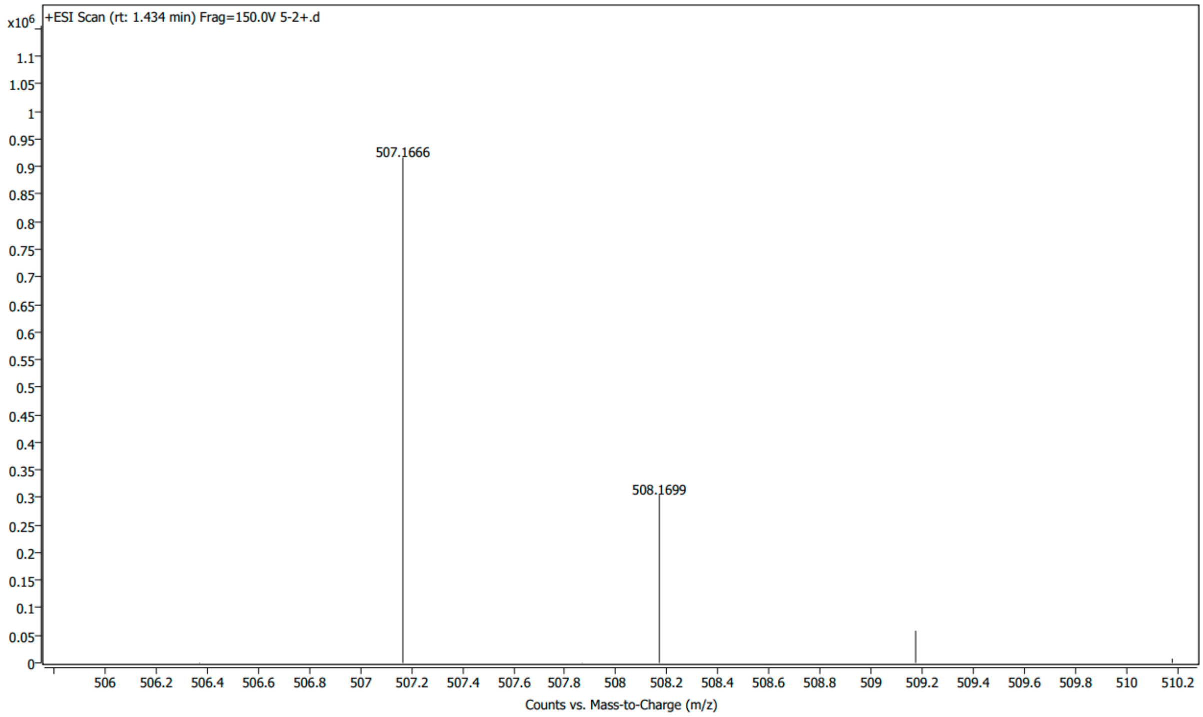

Figure S7 ESI mass spectrum of compound **5** (ESI^{+}).

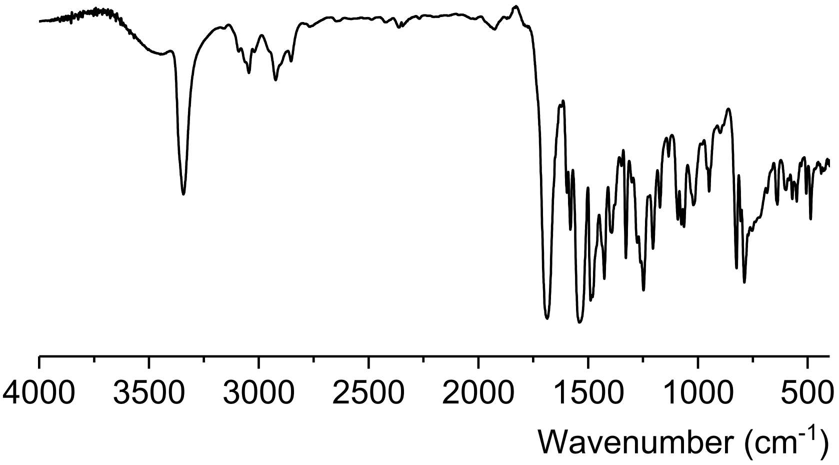

Figure S8 Infrared spectrum of compound **5**.

Figure S9 is the ^{13}C NMR spectrum of **DQBM-B**, Figure S10 is the mass spectrum of **DQBM-B**, which shows a molecular ion peak at

595.2080 corresponding to the $[M+H]^+$ ion of **DQBM-B**.

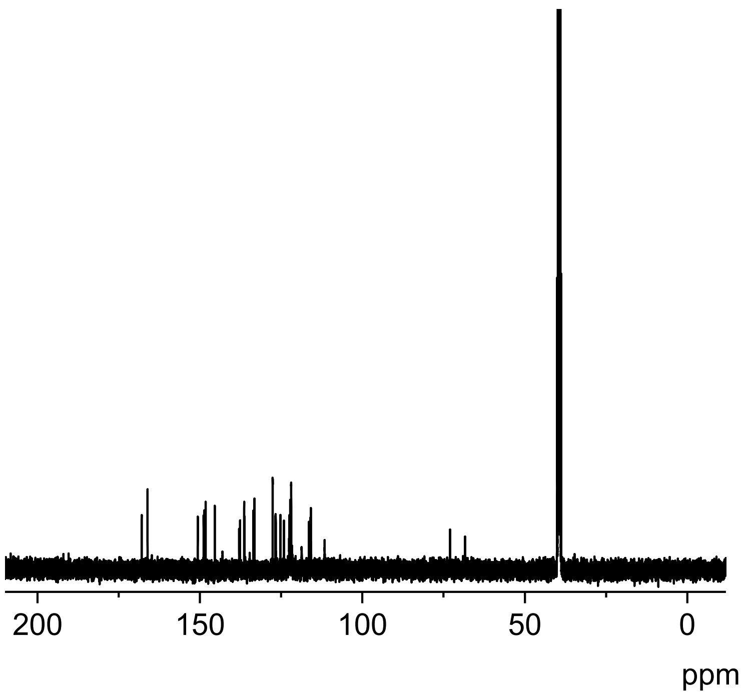

Figure S9 ^{13}C NMR spectrum of DQBM-B.

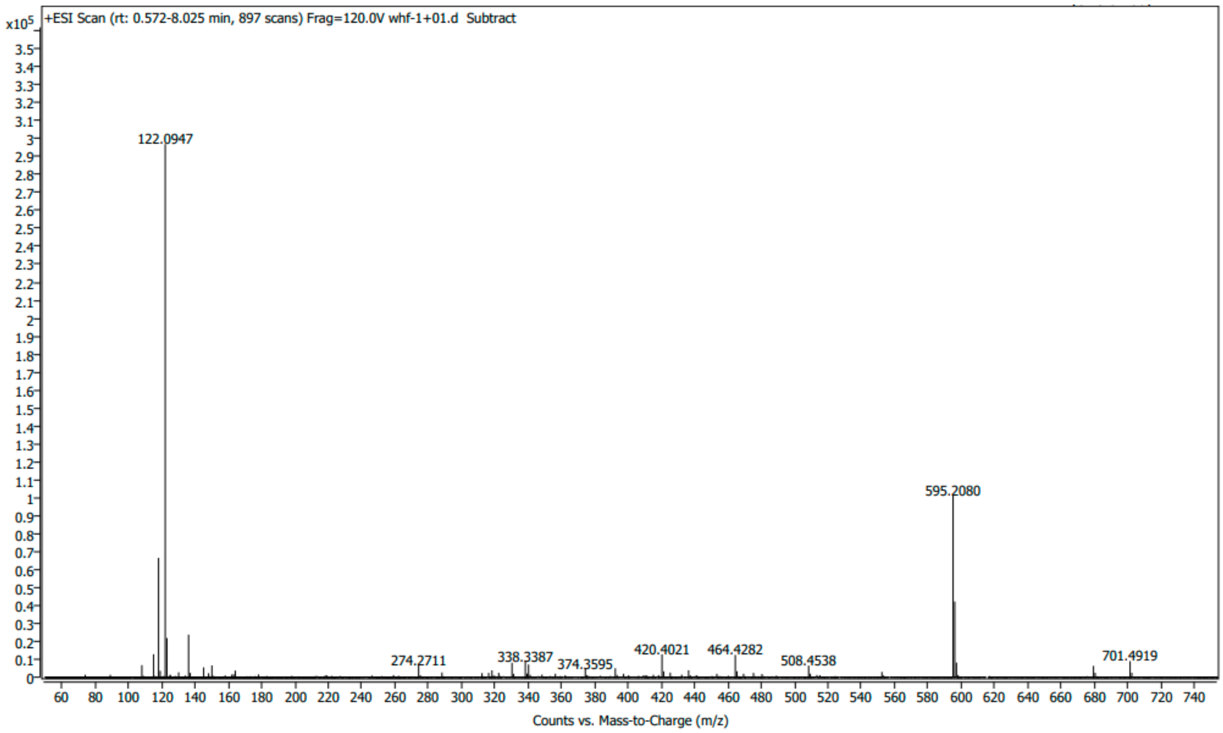

Figure S10 ESI mass spectrum of DQBM-B.
